# Differentiating Liver Metastases from Primary Liver Cancer: A Retrospective Study of Imaging and Pathological Features in Patients with Histopathological Confirmation

**DOI:** 10.3390/biomedicines13010164

**Published:** 2025-01-11

**Authors:** Laura Andreea Ghenciu, Mirela Loredana Grigoras, Luminioara Maria Rosu, Sorin Lucian Bolintineanu, Laurentiu Sima, Octavian Cretu

**Affiliations:** 1 Department of Functional Sciences, Victor Babes University of Medicine and Pharmacy, 300041 Timisoara, Romania; bolintineanu.laura@umft.ro; 2 Department of Anatomy and Embryology, “Victor Babes” University of Medicine and Pharmacy, 300041 Timisoara, Romania; grigoras.mirela@umft.ro (M.L.G.); s.bolintineanu@umft.ro (S.L.B.); 3 Department of Surgical Semiology, Victor Babes University of Medicine and Pharmacy, 300041 Timisoara, Romania; sima.laurentiu@umft.ro (L.S.); octavian.cretu@umft.ro (O.C.)

**Keywords:** liver metastases, primary liver cancer, hepatocellular carcinoma, MRI, imaging features, pathology, differential diagnosis, diagnostic accuracy

## Abstract

**Background and Objectives:** This study aimed to identify and analyze imaging and pathological features that differentiate liver metastases from primary liver cancer in patients with histopathological confirmation, and to evaluate the diagnostic accuracy of imaging modalities. **Materials and Methods:** This retrospective study included 137 patients who underwent liver biopsy or resection between 2016 and 2024, comprising 126 patients with liver metastases and 11 patients with primary liver cancer (hepatocellular carcinoma). Imaging features on contrast-enhanced MRI were evaluated, including lesion number, size, margins, enhancement patterns, presence of capsule, T1/T2 signal characteristics, diffusion-weighted imaging (DWI) signal, and portal vein thrombosis. Laboratory data such as liver function tests and alpha-fetoprotein (AFP) levels were collected. Pathological features recorded included tumor differentiation, vascular invasion, necrosis, and fibrosis. Statistical analyses were performed using chi-squared tests, *t*-tests, and logistic regression, with a significance level of *p* < 0.05. The diagnostic accuracy of imaging features was assessed using receiver operating characteristic (ROC) curve analysis. **Results:** Liver metastases were more likely to present as multiple lesions (82.5% vs. 27.3%, *p* < 0.001), had irregular margins (78.6% vs. 36.4%, *p* = 0.002), rim enhancement (74.6% vs. 18.2%, *p* < 0.001), and were hypointense on T1-weighted images (85.7% vs. 45.5%, *p* = 0.004). Primary liver cancers were more likely to be solitary (72.7% vs. 17.5%, *p* < 0.001), have smooth margins (63.6% vs. 21.4%, *p* = 0.002), exhibit arterial phase hyperenhancement (81.8% vs. 23.8%, *p* < 0.001), and portal venous washout (72.7% vs. 19.0%, *p* < 0.001). Vascular invasion was more common in primary liver cancer (45.5% vs. 11.1%, *p* = 0.01). AFP levels > 400 ng/mL were significantly associated with primary liver cancer (63.6% vs. 4.8%, *p* < 0.001). ROC curve analysis showed that a combination of imaging features had an area under the curve (AUC) of 0.91 for differentiating the two entities. **Conclusions:** Imaging features such as lesion number, margin characteristics, enhancement patterns, T1/T2 signal characteristics, and portal venous washout, along with pathological features like vascular invasion and AFP levels, can effectively differentiate liver metastases from primary liver cancer. The diagnostic accuracy of imaging is high when multiple features are combined.

## 1. Introduction

Liver lesions are commonly encountered in clinical practice, with liver metastases and primary liver cancer being the most prevalent malignant hepatic tumors [1]. Accurate differentiation between liver metastases and primary liver cancer, particularly hepatocellular carcinoma (HCC), is essential for appropriate patient management and prognosis [2]. Magnetic resonance imaging (MRI) is the imaging modality of choice for liver lesion characterization due to its superior soft-tissue contrast and functional imaging capabilities [3].

Liver metastases usually originate from primary tumors such as colorectal, breast, and lung cancers. Their detection often signifies advanced disease and has significant implications for staging and management of the primary malignancy [4]. In contrast, HCC arises from hepatocytes and is commonly associated with chronic liver disease and cirrhosis [5]. Despite advancements in imaging techniques, differentiating liver metastases from HCC remains challenging due to overlapping imaging characteristics [6].

Certain imaging features have been proposed to aid in distinguishing these entities. Liver metastases often present as multiple lesions with rim enhancement, irregular margins, and restricted diffusion, whereas HCC may exhibit arterial phase hyperenhancement, portal venous washout, capsule appearance, and specific signal characteristics on T1- and T2-weighted images [7,8,9]. Pathological examination provides a definitive diagnosis, offering insights into tumor differentiation, vascular invasion, necrosis, and fibrosis [10,11,12].

Additionally, emerging research has delved into the molecular mechanisms that facilitate HCC metastasis, emphasizing the significance of the tumor microenvironment and metabolic regulation in cancer progression. These studies focus on how factors such as cellular signaling pathways and nutrient homeostasis contribute to the metastatic behavior of hepatocellular carcinoma [13,14]. By investigating these underlying processes, researchers aim to identify novel therapeutic targets that can inhibit metastasis and improve clinical outcomes. Integrating these molecular insights with traditional imaging and pathological assessments will enhance our comprehensive understanding of HCC, ultimately leading to more precise diagnostic strategies and the development of targeted treatments tailored to the biological characteristics of each tumor.

Therefore, this study aims to identify specific imaging and pathological features that differentiate liver metastases from primary liver cancer in patients with histopathological confirmation. We also evaluate the diagnostic accuracy of imaging modalities and analyze complex interactions between variables to enhance diagnostic precision.

## 2. Materials and Methods

### 2.1. Study Design and Patient Selection

The current research project was designed as a retrospective study with patients from the Clinical Municipal Hospital from Timisoara, Romania, affiliated with the Victor Babes University of Medicine and Pharmacy from Timisoara, Romania, as well as from local private clinics. This observational study secured ethical approval from the Institutional Review Board, which adheres to the principles set forth in the Declaration of Helsinki [15]. Additionally, this study complies with the EU Good Clinical Practice Directive (2005/28/EC) and the guidelines provided by the International Council for Harmonization of Technical Requirements for Pharmaceuticals for Human Use (ICH), which emphasize informed consent, scientific validity, and the safeguarding of participants’ health and rights.

We conducted a search of the pathology database to identify patients who had undergone liver biopsy or surgical resection between 2016 and 2024. The inclusion criteria specified patients who were 18 years or older, had a histopathological diagnosis of liver metastases or primary liver cancer (hepatocellular carcinoma, HCC), and had undergone preoperative contrast-enhanced MRI of the liver within four weeks prior to their biopsy or surgery. The exclusion criteria ruled out patients who had received previous treatments such as chemotherapy or radiotherapy before imaging, those with poor-quality MRI studies, and cases with incomplete clinical or imaging data. Initially, 152 patients met the inclusion criteria. However, after applying the exclusion criteria, the final analysis included 137 patients, consisting of 126 patients with liver metastases and 11 patients with primary liver cancer.

### 2.2. Imaging Protocol and Evaluation

All MRI studies were conducted using 1.5T or 3.0T scanners and followed standardized liver protocols. The imaging sequences included T1-weighted, T2-weighted, diffusion-weighted imaging (DWI), and dynamic contrast-enhanced sequences, following the administration of gadolinium-based contrast agents. We evaluated several imaging features for each study: the number of lesions (solitary or multiple), the maximum diameter of lesions in centimeters, and their location (right lobe, left lobe, or both), etc. Lesion margins were characterized as either smooth or irregular. Enhancement patterns were scrutinized for arterial phase hyperenhancement, portal venous washout, and rim enhancement, noting the presence or absence of each. Capsule appearance and signal characteristics on T1-weighted images (hypointense, isointense, or hyperintense compared to liver parenchyma) were recorded. Similarly, T2-weighted signal intensities were identified as hyperintense or isointense, and DWI signals were noted for hyperintensity on high b-value images with corresponding low apparent diffusion coefficient (ADC) values [16]. The presence or absence of portal vein thrombosis was also documented.

### 2.3. Laboratory and Pathological Evaluation

Laboratory data were collected within two weeks of imaging, encompassing liver function tests such as Alanine aminotransferase (ALT), aspartate aminotransferase (AST), alkaline phosphatase (ALP), and total bilirubin. Tumor markers were also assessed, including levels of Alpha-fetoprotein (AFP). For pathological evaluation, several features were recorded: tumor differentiation, categorized as well, moderate, or poorly differentiated; vascular invasion; presence or absence of necrosis; and fibrosis, which was graded according to the METAVIR scoring system.

### 2.4. Statistical Analysis

Statistical analyses were conducted using SPSS software version 26.0 (IBM Corp., Armonk, NY, USA). Continuous variables were expressed as mean ± standard deviation, with comparisons made using either the Student’s *t*-test or Mann–Whitney U test, depending on the data distribution. Categorical variables were presented as counts and percentages and analyzed using the chi-squared or Fisher’s exact tests as appropriate. Interobserver agreement was assessed using Cohen’s kappa statistics. Diagnostic accuracy was evaluated through receiver operating characteristic (ROC) curve analysis, and the area under the curve (AUC) values were calculated. For multivariate analysis, logistic regression was performed to identify independent predictors of primary liver cancer, including only variables with a *p* value of less than 0.05 from univariate analysis. A *p* value of less than 0.05 was considered statistically significant.

## 3. Results

Table 1 shows the demographic and clinical characteristics of the study population. Patients with primary liver cancer had a higher prevalence of cirrhosis (63.6% vs. 9.5%, *p* < 0.001) and chronic hepatitis B or C infection (54.5% vs. 7.9%, *p* < 0.001). Serum AFP levels > 400 ng/mL were significantly associated with primary liver cancer (63.6% vs. 4.8%, *p* < 0.001). Liver function tests (ALT, AST, total bilirubin) were elevated in the primary liver cancer group (*p* < 0.001), indicating impaired liver function.

Table 2 presents the imaging features of liver lesions. Liver metastases were more likely to be multiple (82.5% vs. 27.3%, *p* < 0.001) and exhibit rim enhancement (74.6% vs. 18.2%, *p* < 0.001). They commonly had irregular margins (78.6%) and were hypointense on T1-weighted images (85.7%). Primary liver cancers were more likely to be solitary (72.7% vs. 17.5%, *p* < 0.001), have smooth margins (63.6% vs. 21.4%, *p* = 0.002), exhibit arterial phase hyperenhancement (81.8% vs. 23.8%, *p* < 0.001), portal venous washout (72.7% vs. 19.0%, *p* < 0.001), capsule appearance (54.5% vs. 11.1%, *p* < 0.001), and portal vein thrombosis (45.5% vs. 4.8%, *p* < 0.001). Hyperintensity on T2-weighted images was more frequent in primary liver cancer (81.8% vs. 57.1%, *p* = 0.03).

Table 3 shows the pathological features of liver lesions. Vascular invasion was more common in primary liver cancer (45.5% vs. 11.1%, *p* = 0.01). Fibrosis stages F2–F4 (indicative of significant fibrosis or cirrhosis) were significantly more prevalent in primary liver cancer (72.7% vs. 9.5%, *p* < 0.001). Tumor differentiation and the presence of necrosis did not differ significantly between the groups.

Table 4 summarizes the diagnostic performance of various imaging features in assessing a specific condition, including their sensitivity, specificity, Area Under the Curve (AUC), and associated *p*-values. The sensitivity of multiple lesions is 82.5% with a specificity of 72.7% and an AUC of 0.77, all statistically significant with a *p*-value of less than 0.001. Arterial Phase Hyperenhancement shows slightly lower sensitivity at 81.8% but higher specificity at 76.2%, and an AUC of 0.79. Portal Venous Washout has a sensitivity of 72.7% and a specificity of 81%, with an AUC also at 0.77. Rim Enhancement’s sensitivity and specificity are 74.6% and 81.8%, respectively, with an AUC of 0.78. Capsule Appearance shows a lower sensitivity of 54.5% but a high specificity of 88.9%, with an AUC of 0.72. Portal Vein Thrombosis presents the lowest sensitivity at 45.5% but the highest specificity at 95.2%, and an AUC of 0.7. The combined imaging features significantly enhance diagnostic accuracy, showing a sensitivity of 90.9% and specificity of 85.7%, with the highest AUC of 0.91, all with statistically significant outcomes (*p*-value < 0.001).

The analysis indicates that cirrhosis significantly increases the odds of the outcome, with an adjusted odds ratio (OR) of 10.5 and a confidence interval (CI) ranging from 2.8 to 39.4, and a *p*-value of less than 0.001. High levels of AFP (>400 ng/mL) are associated with an OR of 8.2 (CI: 2.1–32.1) and a *p*-value of 0.003. Arterial phase hyperenhancement, portal venous washout, and portal vein thrombosis also significantly affect the outcome with ORs of 6.7, 5.5, and 4.9, respectively, and corresponding *p*-values indicating statistical significance. Conversely, having multiple lesions is associated with a decreased odds of the outcome, reflected by an OR of 0.2 and a statistically significant *p*-value of 0.015 (Table 5).

The agreement on “Lesion Number” was particularly strong, with a kappa value of 0.92, suggesting near-perfect consistency. Other features such as “Margin Characteristics”, “Enhancement Patterns”, “Capsule Appearance”, and “Portal Vein Thrombosis” also showed high levels of agreement, with kappa values ranging from 0.78 to 0.89 (Table 6).

For the multivariate logistic regression model, the variables Cirrhosis, AFP > 400 ng/mL, Arterial Phase Hyperenhancement, Portal Venous Washout, Portal Vein Thrombosis, and Multiple Lesions were included in the multivariate analysis, as these factors were statistically significant (*p* < 0.05) in the univariate analysis. This selection ensures the model’s accuracy and reproducibility by focusing on the most relevant predictors of primary liver cancer. Of the 19 cirrhotic patients, 36.8% had primary liver cancer, compared to only 3.4% of the 118 non-cirrhotic patients, with a statistically significant difference (*p*-value < 0.001). Similarly, high AFP levels (>400 ng/mL) were observed in 47.4% of cirrhotic patients vs. 3.4% of non-cirrhotic patients, also showing a significant difference (*p*-value < 0.001). Liver metastases were more common in non-cirrhotic patients (96.6%) compared to cirrhotic ones (63.2%). Other imaging features like arterial hyperenhancement and portal venous washout were more frequently observed in cirrhotic patients, with respective *p*-values of 0.015 and 0.04. Capsule appearance was significantly different between the two groups, with 47.4% of cirrhotic patients showing this feature compared to only 9.3% of non-cirrhotic patients (*p*-value < 0.001), as seen in Table 7.

## 4. Discussion

### 4.1. Important Findings and Literature Review

This study contributes to enhancing the accuracy of differentiating hepatic lesions, and confirms that distinct imaging and pathological characteristics can effectively distinguish between liver metastases and primary liver cancer. The differences in imaging features between these two conditions highlight their disparate origins and growth behaviors, aiding in accurate diagnosis. In terms of lesion number and margin characteristics, liver metastases typically manifest as multiple lesions with irregular margins, reflecting their metastatic origin and invasive growth patterns [17,18,19]. In contrast, hepatocellular carcinoma usually presents as a single lesion with smooth margins and often features a capsule, which indicates its development from hepatocytes and the propensity to generate a pseudocapsule [20]. These distinctions are crucial for differentiating between the two types of liver malignancies in clinical settings.

Moreover, the enhancement patterns observed during imaging further support this differentiation. HCC is characterized by arterial phase hyperenhancement and rapid portal venous washout due to its predominant arterial blood supply [21]. Conversely, rim enhancement, indicative of peripheral neovascularization and central necrosis, is more commonly seen in metastases [22]. Additionally, HCC may show variable signal intensities on T1- and T2-weighted images and is more likely to be associated with portal vein thrombosis, a factor linked with a poorer prognosis. These imaging features provide essential clues for the diagnosis and assessment of liver tumors.

Pathological and diagnostic features significantly impact the differentiation between liver metastases and hepatocellular carcinoma. There is a well-documented strong association between cirrhosis and HCC, with significant fibrosis stages (F2–F4) more prevalent among patients with primary liver cancer [23,24]. Furthermore, vascular invasion, a hallmark of aggressive HCC, correlates with higher risks of metastasis and recurrence, while elevated alpha-fetoprotein levels, although not entirely specific, serve as a recognized marker for HCC. These characteristics underscore the critical nature of accurate pathological assessment in diagnosing liver cancer.

In a similar manner, Ozaki et al. [25] focused on the variability in imaging features of liver metastases across different primary cancers. They described specific imaging characteristics, such as the target sign on T2-weighted MR images and peritumor hyperintensity, which could aid in more precise diagnosis, especially in complex clinical scenarios like unknown primary tumors or multiple malignancies. Moreover, Bohlok et al. [26] found that the histopathological growth pattern (HGP) of liver metastases serves as an independent marker for metastatic behavior across various primary cancers. They examined a large cohort of patients with colorectal (N = 263) and non-colorectal (N = 66) liver metastases, identifying significant survival differences based on HGP. Patients with a desmoplastic HGP exhibited notably better outcomes, with a 5-year overall survival of 57% compared to 41% in those with a non-desmoplastic HGP. This histological feature proved to be a more significant predictor of survival than traditional risk factors, emphasizing its potential as a clinical tool for assessing prognosis following surgical resection.

Additionally, Huang et al. [27] analyzed 156 patients, finding that larger tumor diameters, irregular margins, the presence of intratumoral vessels, and peritumoral hypointensity during the hepatobiliary phase are significant markers of high-grade HCC, with the maximum tumor diameter showing an odds ratio of 1.002 as an independent risk factor. In a parallel investigation, Gigante et al. [28] studied 212 patients and identified non-smooth tumor margins and the macro-trabecular massive histological subtype as strong predictors of aggressive intrasegmental recurrence (AIR) after radiofrequency ablation, with hazard ratios of 3.7 and 3.8, respectively. These studies highlight that specific preoperative imaging and histological markers not only stratify patients by risk but also guide more personalized therapeutic decisions for HCC treatment.

Considering our findings, Hayano et al. [29] assessed the diagnostic value of computed tomography perfusion (CTP) in differentiating HCC from metastatic liver tumors, analyzing CTP data from 90 liver tumors. Their findings revealed that hypovascular metastases exhibited significantly lower blood flow (BF) and blood volume (BV), and higher mean transit time (MTT) compared to HCC. Conversely, the values of BF, BV, and MTT for HCC were substantially lower than those of hypervascular metastases, identifying BV as a useful marker in distinguishing HCC from hypervascular metastases through receiver-operating characteristic analysis.

In a similar manner, the study by Fabritius et al. [30] evaluated the diagnostic accuracy of somatostatin receptor-positron emission tomography/computed tomography (SSR-PET/CT) in identifying liver metastases from well-differentiated neuroendocrine tumors (NETs) against histopathology, which is the reference standard. They found that SSR-PET/CT showed a positive predictive value of 91.0% in diagnosing liver metastases of NET, which improved to 95.5% after re-biopsy of initially negative lesions. This highlights SSR-PET/CT’s high diagnostic accuracy, though it noted that about 4–5% of G2 NETs, with a Ki-67 index between 2 and 15%, did not show SSR uptake, suggesting a potential need for complementary imaging techniques like [18F]FDG PET/CT in certain NET cases.

Hatzidakis et al. [31] analyzed the efficacy of various CT liver perfusion (CTLP) parametric maps across 26 patients with 50 HCC lesions, identifying the maximum slope of increase (MSI) as the most effective parameter with a sensitivity of 96% and specificity of 100% for distinguishing HCC from non-tumorous parenchyma. The MSI showed a remarkable area under the ROC curve of 0.997, using a cut-off of 2.2 HU/s. In a similar manner, Fischer et al. [32] evaluated dynamic perfusion CT (P-CT) in 26 cirrhotic patients, finding that hepatic perfusion-index (HPI) maps, especially when combined with arterial maximum intensity projections (art-MIP), significantly enhanced HCC detection rates with sensitivity and specificity values reaching up to 100% at certain cut-off thresholds.

The studies by Mocan et al. [33] and Zhang et al. [34] explore the diagnostic and prognostic potentials of immunohistochemistry and imaging in liver cancers, respectively. Mocan et al. focused on the differentiation between intrahepatic cholangiocarcinoma (iCCA), HCC, and liver metastases using immunohistochemical stains. They identified CK19 and CA19-9 as highly sensitive markers for iCCA, and Glypican 3 and Hep Par 1 for HCC, with sensitivities reaching 100% in detecting these cancers. Furthermore, they observed that CK7 expression and the amount of intratumoral immune cells were significant prognostic factors for overall survival. In a similar manner, Zhang et al. assessed the preoperative prediction of histological grade and microvascular invasion (MVI) in HCC patients using MRI features. They found significant correlations between multiple lesions and high-grade or MVI-positive HCC, with specific MRI signs such as artistic rim enhancement and tumor margin also showing statistical significance in predicting MVI presence.

The diagnostic accuracy improves substantially when multiple imaging features are combined, achieving an AUC of 0.91. This enhancement emphasizes the importance of a comprehensive imaging evaluation over-reliance on a single feature. Clinically, precise differentiation between liver metastases and HCC is paramount for appropriate patient management. Misclassification can result in incorrect treatment approaches, such as undue systemic chemotherapy for metastases or overlooked opportunities for potentially curative treatments in cases of HCC, highlighting the need for meticulous and informed diagnostic processes.

While our study demonstrated that elevated AFP levels are a strong marker for distinguishing primary liver cancer from liver metastases, we did not evaluate the dynamic changes in AFP levels during treatment and their potential correlation with disease prognosis. Understanding how AFP levels fluctuate in response to therapy could provide valuable insights into treatment efficacy and patient outcomes. Future research should focus on longitudinal monitoring of AFP to assess its prognostic value and its ability to predict recurrence or survival rates. Incorporating such dynamic assessments could enhance the clinical utility of AFP as not only a diagnostic biomarker but also as a tool for ongoing disease management and prognostication.

These findings provide crucial insights into differentiating liver metastases from primary liver cancer through distinct imaging and pathological features. By identifying key indicators such as the prevalence of cirrhosis, elevated AFP levels, and specific MRI characteristics like arterial phase hyperenhancement and portal venous washout, clinicians can achieve more accurate and timely diagnoses. This differentiation is vital for tailoring appropriate treatment strategies, which can lead to improved patient outcomes and more efficient allocation of healthcare resources. Additionally, the ability to accurately distinguish between these liver conditions facilitates better prognostic assessments and enables the implementation of targeted therapeutic interventions, ultimately enhancing the overall management of patients with liver malignancies.

However, the limitations of our study, including the small sample size of primary liver cancer cases and the exclusive use of MRI as the imaging modality, have implications for the robustness and generalizability of our conclusions. The limited number of primary liver cancer cases may reduce the statistical power and increase the potential for type II errors, thereby affecting the reliability of the observed associations. Moreover, relying solely on MRI restricts the applicability of our findings to clinical settings where other imaging modalities, such as CT or contrast-enhanced ultrasound, are available and commonly used. These constraints necessitated our methodological choices, as we utilized the best available resources within our institution to conduct a comprehensive analysis.

### 4.2. Study Limitations

This study is constrained by several limitations, including a small sample size of primary liver cancer cases, which may affect the statistical power and the generalizability of the findings. Its retrospective design could introduce selection bias and result in incomplete clinical or imaging data. Being a single-center study, the findings might not extend to other institutions that have different patient demographics or imaging protocols. Additionally, the study’s exclusive focus on MRI data and the lack of inclusion of other imaging modalities such as contrast-enhanced ultrasound or CT scans might have restricted the scope for gathering more comprehensive insights. Finally, the reliance on diagnostic accuracy metrics such as AUC without adequately addressing real-world applicability, including the potential impacts of false positives and false negatives.

## 5. Conclusions

Imaging features such as lesion number, margin characteristics, enhancement patterns, T1/T2 signal characteristics, and portal venous washout, along with pathological features like vascular invasion, cirrhosis, and elevated AFP levels, can effectively differentiate liver metastases from primary liver cancer. The diagnostic accuracy of imaging is high when multiple features are combined. Recognizing these features can guide clinicians in accurate diagnosis and appropriate management strategies, ultimately improving patient outcomes.

## Figures and Tables

**Table 1:** Patient Demographics and Clinical Characteristics.

Characteristic	Liver Metastases (n = 126)	Primary Liver Cancer (n = 11)	*p*-Value
Age (years)	58.4 ± 10.2	62.7 ± 9.5	0.12
Male sex	74 (58.7%)	8 (72.7%)	0.35
Body Mass Index (BMI)	26.1 ± 4.5	27.3 ± 4.8	0.39
Cirrhosis	12 (9.5%)	7 (63.6%)	<0.001
Chronic Hepatitis B/C	10 (7.9%)	6 (54.5%)	<0.001
AFP > 400 ng/mL	6 (4.8%)	7 (63.6%)	<0.001
ALT (U/L)	35.2 ± 15.6	72.5 ± 30.1	<0.001
AST (U/L)	40.1 ± 18.2	80.3 ± 35.4	<0.001
ALP (U/L)	98.6 ± 40.2	120.5 ± 50.3	0.08
Total Bilirubin (mg/dL)	1.1 ± 0.5	2.3 ± 1.0	<0.001

**Table 2:** Imaging Features of Liver Lesions.

Characteristic	Liver Metastases (n = 126)	Primary Liver Cancer (n = 11)	*p*-Value
Lesion Number			<0.001
Solitary	22 (17.5%)	8 (72.7%)	
Multiple	104 (82.5%)	3 (27.3%)	
Lesion Size (cm)	3.2 ± 1.5	4.8 ± 2.0	0.01
Lesion Location			0.02
Right Lobe	86 (68.3%)	9 (81.8%)	
Left Lobe	24 (19.0%)	1 (9.1%)	
Both Lobes	16 (12.7%)	1 (9.1%)	
Margin Characteristics			0.002
Smooth	27 (21.4%)	7 (63.6%)	
Irregular	99 (78.6%)	4 (36.4%)	
Enhancement Patterns			<0.001
Arterial Phase Hyperenhancement	30 (23.8%)	9 (81.8%)	
Portal Venous Washout	24 (19.0%)	8 (72.7%)	
Rim Enhancement	94 (74.6%)	2 (18.2%)	
Capsule Appearance	14 (11.1%)	6 (54.5%)	<0.001
T1-Weighted Signal			0.004
Hypointense	108 (85.7%)	5 (45.5%)	
Isointense	12 (9.5%)	4 (36.4%)	
Hyperintense	6 (4.8%)	2 (18.2%)	
T2-Weighted Signal			0.03
Hyperintense	72 (57.1%)	9 (81.8%)	
Isointense	54 (42.9%)	2 (18.2%)	
DWI Hyperintensity	112 (88.9%)	10 (90.9%)	0.99
Portal Vein Thrombosis	6 (4.8%)	5 (45.5%)	<0.001

**Table 3:** Pathological Features of Liver Lesions.

Pathological Features	Liver Metastases (n = 126)	Primary Liver Cancer (n = 11)	*p*-Value
Tumor Differentiation		0.15	
Well Differentiated	20 (15.9%)	2 (18.2%)	
Moderately Differentiated	75 (59.5%)	7 (63.6%)	
Poorly Differentiated	31 (24.6%)	2 (18.2%)	
Vascular Invasion	14 (11.1%)	5 (45.5%)	0.01
Necrosis	68 (54.0%)	6 (54.5%)	0.97
Fibrosis (METAVIR Score)		<0.001	
F0–F1	114 (90.5%)	3 (27.3%)	
F2–F4	12 (9.5%)	8 (72.7%)	

**Table 4:** Diagnostic Performance of Imaging Features.

Imaging Feature	Sensitivity (%)	Specificity (%)	AUC	*p*-Value
Multiple Lesions	82.5	72.7	0.77	<0.001
Arterial Phase Hyperenhancement	81.8	76.2	0.79	<0.001
Portal Venous Washout	72.7	81	0.77	<0.001
Rim Enhancement	74.6	81.8	0.78	<0.001
Capsule Appearance	54.5	88.9	0.72	<0.001
Portal Vein Thrombosis	45.5	95.2	0.7	<0.001
Combined Imaging Features	90.9	85.7	0.91	<0.001

**Table 5:** Multivariate Logistic Regression Analysis.

Variable	Adjusted OR (95% CI)	*p*-Value
Cirrhosis	10.5 (2.8–39.4)	<0.001
AFP > 400 ng/mL	8.2 (2.1–32.1)	0.003
Arterial Phase Hyperenhancement	6.7 (1.7–26.1)	0.006
Portal Venous Washout	5.5 (1.4–21.7)	0.015
Portal Vein Thrombosis	4.9 (1.1–21.4)	0.037
Multiple Lesions	0.2 (0.05–0.7)	0.015

**Table 6:** Interobserver Agreement for Imaging Features.

Imaging Feature	Cohen’s Kappa Value	*p*-Value
Lesion Number	0.92	<0.001
Margin Characteristics	0.85	<0.001
Enhancement Patterns	0.78	<0.001
Capsule Appearance	0.81	<0.001
Portal Vein Thrombosis	0.89	<0.001

**Table 7:** Subgroup Analysis in Cirrhotic and Non-Cirrhotic Patients.

Variable	Cirrhotic Patients (n = 19)	Non-Cirrhotic Patients (n = 118)	*p*-Value
Primary Liver Cancer	7 (36.8%)	4 (3.4%)	<0.001
Liver Metastases	12 (63.2%)	114 (96.6%)	
AFP > 400 ng/mL	9 (47.4%)	4 (3.4%)	<0.001
Arterial Hyperenhancement	10 (52.6%)	29 (24.6%)	0.015
Portal Venous Washout	8 (42.1%)	24 (20.3%)	0.04
Capsule Appearance	9 (47.4%)	11 (9.3%)	<0.001

## Data Availability

The data presented in this study are available on request from the corresponding author.
